# Wash-free Imaging in Live Cells

**DOI:** 10.1021/acscentsci.4c02083

**Published:** 2025-01-06

**Authors:** Mahdi Hasan, Ashraf Brik

**Affiliations:** Schulich Faculty of Chemistry Technion-Israel Institute of Technology Haifa, 3200008, Israel

In this issue of *ACS Central Science*, Jbara, Vendrell, and co-workers report the development of a powerful mini-labeling approach to track the cellular uptake routes of peptides and proteins.^1^ Cell delivery of custom-made peptides/proteins and other biomolecules is of great interest for basic research and therapeutic applications.^2^ Investigating the entry mechanism and tracking the path of delivered biomolecules to their specific organelle and target are extremely challenging and require tools yet to be further developed and optimized for each target. In this context, fluorescence microscopy has been widely used for studying peptide/protein localization and trafficking with high spatial and temporal resolution. Therefore, great efforts have been invested to find the most effective labeling method that would 1) allow for high-resolution microscopy, also termed super-resolution microscopy (SR); 2) enable wash-free microscopy experiments for real-time tracking; 3) not interfere with the activity of the labeled biomolecule; and 4) not affect the delivery mechanism. Despite some advances in this area and the development of dyes, e.g., cyanines and rhodamines, we still lack a method that can fulfill all requirements. A dye that is neutrally charged and as small as an amino acid side chain will have clear advantages. Regarding the mechanism of action of some of these dyes, point accumulation for imaging in nanoscale topography (PAINT), which has been gaining great interest, utilizes fluorophores that emit light upon binding to targets, with early reports focusing on lipophilic dyes that fluoresce strongly in hydrophobic environments.^3^

**Figure 1:**
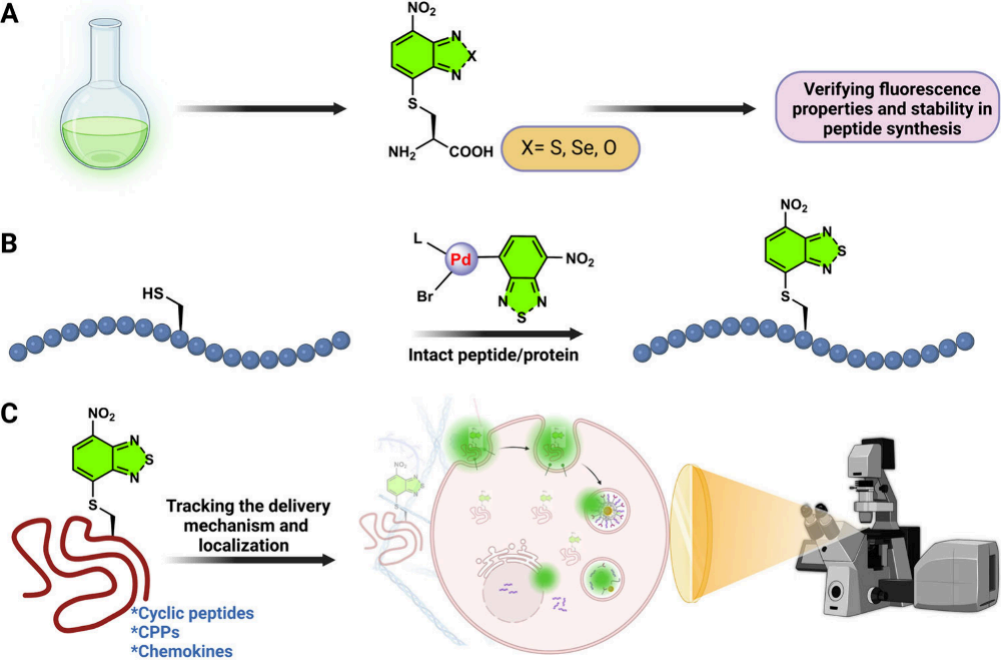
(A) Cys-linked benzodiazole derivatives for wash-free imaging. (B) Modification of unprotected peptides or proteins via palladium-mediated arylation. (C) Tracking the delivery mechanism and localization of the labeled biomolecule using fluorescence microscopy in live cells.

To further move this field forward and prepare optimal probes for real-time imaging, the Vendrell group previously developed small unnatural fluorescent amino acids and integrated them into peptides to generate live-cell imaging reporters. The researchers have screened several benzodiazole derivatives, containing C, O, S, or Se in the heterocycle component, attached via S- or N-linkage to the β-carbon of the amino acid. They examined their suitability as reporters for wash-free fluorescence microscopy and incorporation in solid-phase peptide synthesis (SPPS, Figure 1A). These studies have shown that the S-linked amino acid with the thiol-containing heterocycle has the most desirable fluorescence properties, concerning photostability, exhibiting a low background signal in aqueous media and a strong fluorogenic response in a nonpolar environment. Yet when incorporated in a peptide via SPPS, it showed acid lability, rendering it unsuitable for peptide synthesis.^4^

In the current issue of *ACS Central Science*, Jbara, Vendrell, and co-workers presented a creative solution to this problem and were able to efficiently incorporate the Cys-linked benzodiazole as a small fluorescent molecule into peptides and proteins without affecting their bioactivity and their delivery mechanism. The authors developed an organometallic palladium complex bearing a benzodiazole moiety for site-selective insertion of the fluorogenic probe into unprotected peptides or proteins bearing a free Cys residue through palladium-mediated S-arylation,^5^ bypassing the exposure of this amino to peptide synthesis conditions (Figure 1B). With this late-stage modification approach, the authors successfully assessed the fluorescence emission of three Cys-benzodiazole derivatives, containing O, S, or Se in the heterocycle component. This study revealed that the S-analog exhibited up to a 27-fold fluorescence increase, with the lowest background signals in water and the strongest response in hydrophobic conditions.

The authors demonstrated their method for labeling and tracking different cell-penetrating peptides (CPPs). Specifically, they modified TAT, Penetratin, and sC18 with benzo-2,1,3-thiadiazole. Following wash-free imaging experiments in live cells, they determined the delivery mechanism of the CPPs in spatiotemporal resolution. Once the CPP starts internalizing the cell, the hydrophobic surrounding enables the fluorescence emission of the benzodiazole to rise dramatically, allowing tracking of the delivery mechanisms in live-time imaging (Figure 1C). Their results showed that the TAT peptide internalized the cell via direct translocation, Penetratin via micelle-mediated internalization and endocytic uptake, while the sC18 internalized the cell via endocytic uptake, all of which agree with previous reports. Notably, for the sC18 peptide, the authors performed a fluorescence lifetime imaging microscopy (FLIM) experiment in live cells. They analyzed the florescent lifetime of the peptide in a localization-dependent manner, indicating that sC18 has a longer lifetime when interacting with the plasma membrane and a shorter lifetime in the cytosol and after translocation to the nucleus. The authors observed rapid nuclear localization of the sC18, indicating that it may have a dual internalization mechanism involving cellular endocytosis and direct translocation. Finally, the authors successfully labeled the expressed chemokine protein (mCCL2), bearing two disulfide bonds and a free Cys residue without distributing the native S–S bonds. This enabled mCCL2 tracking in live cells, demonstrating its binding to the chemokine receptor CCR2 and the endocytosis trafficking mechanism.

Despite demonstrating the effectiveness of this approach in labeling different peptides, it still has some limitations. Labeling Cys-rich peptides and proteins using palladium-mediated arylation will be challenging and may affect the homogeneity of the product and its function. In principle, this could be overcome by using several technologies for modifying intact proteins and peptides such as π-clump,^6^ sortase,^7^ coiled-coil PNA labeling,^8^ and palladium-mediated S-arylation on metal-binding motifs.^9^ Additionally, genetic code expansion could allow precise and selective preparation of modified proteins with benzodiazole.^10^

The powerful method developed by Jbara and Vendrell allows for rapidly accessing modified peptides, proteins, and possibly other biomolecules, with minimal labeling. This lays the foundation for 1) investigating delivery and trafficking mechanisms in spatiotemporal resolution; 2) deciphering the role of several cytokines in immune responses and cell signaling; and 3) studying the effect of post-translationally modified proteins on their dynamics in live cells. By further expanding the labeling toolkit of proteins with the benzodiazole moiety, using the above proposed methods, one could access many labeled proteins and address several questions related to their journey in biological processes.
